# *Actinotignum schaalii* infection: Challenges in diagnosis and treatment

**DOI:** 10.1016/j.heliyon.2024.e28589

**Published:** 2024-03-22

**Authors:** J.M. Sahuquillo-Arce, P. Suárez-Urquiza, A. Hernández-Cabezas, L. Tofan, R. Chouman-Arcas, M. García-Hita, O. Sabalza-Baztán, A. Sellés-Sánchez, N. Lozano-Rodríguez, J. Martí-Cuñat, J.L. López-Hontangas

**Affiliations:** a Servicio de Microbiología, Hospital Doctor Moliner, Serra, Spain; b Servicio de Microbiología, Hospital Universitari i Politècnic La Fe, Valencia, Spain; c Servicio de Análisis Clínicos, Hospital Universitari i Politècnic La Fe, Valencia, Spain; d Servicio de Microbiología, Hospital Francesc de Borja, Gandia, Spain; e Servicio de Microbiología, Hospital de la Vega Lorenzo Guirao, Cieza, Spain; f Servicio de Microbiología, Hospital General Universitari de Castelló, Castellón de la Plana, Spain; g Hospital General Universitario Santa Lucía, Cartagena, Spain; h Grupo de Investigación de Infecciones Respiratorias, IIS La Fe, Valencia, Spain

**Keywords:** *Actinotignum schaalii*, Isolation, Antimicrobial resistance, Epidemiology

## Abstract

*Actinotignum schaalii* affects elderly people and is associated with individuals with urological-related predispositions, but can be found in a variety of locations, such as cutaneous, intraabdominal, genitourinary and surgical infections. Disseminated infections occur less frequently and are by and large related to urinary tract colonisation.

This pathogen is often neglected due to growth requirements, especially in urinary tract infections. We present 107 *Actinotignum schaalii* isolated from genitourinary samples (80.4%), from skin and soft tissue infections (13.1%), from bone and deep tissue infection (4.7%) and from blood cultures (1.9%). The automated system Alfred 60/AST was paramount for the isolation of 77.6% of the UTI.

All the isolates tested were susceptible to penicillin, ampicillin, linezolid, vancomycin, teicoplanin, rifampicin and tetracycline.

In conclusion, we present a large series of *Actinotignum schaalii* infections. This pathogen is hard to isolate, and is resistant to commonly used empirical antimicrobials.

## 1 Introduction

The most relevant species for humans within the genus *Actinotignum*, which belongs to the *Actinomycetaceae* family, is *Actinotignum schaalii*, primarily associated with urinary tract infections (UTI). It was initially described within the genus *Actinobaculum* by Lawson et al., in 1997 [1] but later, in 2015, Yassin et al. proposed a reclassification of the species in a new genus, *Actinotignum* [2].

*A. schaalii* is a small, facultative anaerobic, non-motile, non-spore forming, non-acid-alcohol resistant, slightly curved, Gram positive coccoid rod. It displays slow, fastidious growth and requires 48–72h in blood agar-enriched media in an anaerobic or 5% CO_2_ atmosphere. In solid media, it grows as small greyish colonies with weak or absent alpha-haemolysis [3]. Thus, growth characteristics and requirements mean *A. schaalii* goes unnoticed very often, chiefly in UTI where it is frequently underdiagnosed.

The natural habitat of this microorganism is not exactly known but it is probably a commensal bacterium of the urogenital tract in humans. Nielsen et al. found DNA from *A. schaalii* in 22% of patients aged 60 or older, with counts between 10^4 and 10^7/mL [4]. It can also be found in children younger than 4 years old and this colonisation could be explained by the moist environment created by nappies [5]. Olsen et al. analysed both faecal and vaginal swabs in patients with no urological disease and *A. schaalii* genetic material was absent in all faecal samples; however, it was detected in 32% of the vaginal swabs [6]. The latter supports the claim that *A. schaalii* is part of the urogenital microbiome. Moreover, whole genome sequencing revealed fimbria genes in *A. schaalii* that would favour the colonisation of the urinary tract [7].

*A. schaalii* mostly affects elderly people and is associated with individuals with urological-related predispositions, such as bladder or prostate cancer, urinary incontinence, catheterisation, prostate hyperplasia, neurogenic bladder, urethral stenosis and chronic renal failure, as well as immunosuppression [4,8]. Additionally, it can be found in abscesses in a variety of locations, such as cutaneous, intraabdominal, genitourinary and surgical infections [9,10]. Disseminated infections including sepsis, spondylodiscitis, bacteraemia and endocarditis occur less frequently and are related to urinary tract colonisation since most patients present some kind of urological disease [[11], [12], [13], [14], [15]].

Because of growth requirements, this pathogen is often neglected, especially in UTI, because standard practice for doing urine cultures does not include incubation in a CO_2_-enriched atmosphere. Furthermore, many labs use chromogenic media like CPSE that do not support growth of most strains of *A. schaalii*. Moreover, basic urine screening tests fall short as results from the urine strip test are negative due to the lack of nitrate reductase activity. Phenotypic testing is time-consuming and inadequate; thus, nucleic acids amplification (PCR), sequencing or MALDI-ToF are needed [16]. PCR techniques have the disadvantage of being too sensitive, detecting the pathogen in asymptomatic cases [5]. MALDI-ToF, on the other hand, is performed on cultured isolates and prevents misdiagnosis in colonised patients [16].

Additionally, empirical treatment can fail since *A. schaalii* is resistant to trimethoprim and ciprofloxacin, both widely used therapeutic options for the treatment of UTI. Therefore, isolation of the pathogen and antimicrobial susceptibility testing are essential for correct treatment, but also to glean the real magnitude of infections caused by *A. schaalii*, especially UTI [4,5,[8], [9], [10],^12^,^13^,^15^,[17], [18], [19]].

The objective of this study was to analyse *A. schaalii* infections at Hospital Universitari i Politècnic La Fe, regarding the isolation site, type of patient, susceptibility profile and pathogen isolation.

## 2 Material and methods

This is a retrospective study of infections cause by *A. schaalii* in the health department of Hospital Universitari i Politècnic La Fe in Valencia, Comunitat Valenciana, Spain between 2016 and 2021.

Data on *A. schaalii* and UTI by other microorganisms were obtained from the laboratory management software tool Cointec Gestlab© and processed with Microsoft Excel© and SPSS IBM© v.21.0. The Mann-Whitney *U* test and χ^2^ test were employed for the statistical analysis. A random sample of 120 patients was selected from the database to determine the prevalence of urogenital anomalies in UTI. Repeated urinary isolates from the same patient in less than 21 days were considered as the same infectious episode and were therefore eliminated from the database.

### 2.1 Isolates and laboratory procedures

About two thirds of the urine cultures in our laboratory are performed by the fully automated bacterial culture system, Alfred 60/AST, Alifax©, where a patient's urine sample is inoculated in a liquid enriched culture broth and incubated in vortex agitation for 4 h. Changes in the media diffraction are analysed by a laser system with two angles (30° and 90°) to detect growth; and, if detected, samples are inoculated in chromogenic media CPSE (BioMérieux, Marcy l’étoile) and incubated for 24 h. If by this time the solid culture media shows no growth, the sample is reincubated in a CO_2_ atmosphere for 24 more hours and a Gram stain of the urine sample is performed. If Gram positive rods are observed, the sample is inoculated in Columbia agar in a CO_2_ atmosphere. Although some strains of *A. schaalii* may grow in CPSE if incubated in a CO_2_ atmosphere, the original CPSE inoculated by Alfred 60/AST (Alifax©) has alredy been 24 h in ambient air; thus, a new subculture in Columbia agar is preferable. The remaining third of the urine samples are manually inoculated in CPSE or CHROMagar™ Orientation (Becton-Dickinson, Franklin Lakes). Non-urine samples are cultured according to laboratory procedures based on international microbiology guides.

Isolates were identified with Matrix Assisted Laser Desorption/ionisation-Time of Flight (MALDI-ToF) Vitek MS® (BioMérieux, Marcy l′étoile) or microflex® LRF (Bruker, Billerica).

### 2.2 Antimicrobial susceptibility

Antimicrobial susceptibility was determined by the disc-diffusion method in Columbia agar in a CO_2_ atmosphere for 24–48 h. Due to the lack of standardised criteria for the *Actinotignum* genus, susceptibility was interpreted by EUCAST criteria for *Corynebacterium*. Epsilon tests (BioMérieux, Marcy l′étoile) were used for antimicrobials that lacked EUCAST breakpoints, i.e. nitrofurantoin and fosfomycin, and the MIC50 and MIC90 were calculated.

### 2.3 Colony Forming Units (CFU)/mL detection assay

*A. schaalii* detection by Alfred 60/AST Alifax© was assessed through serial dilution of 20 randomly selected strains in concentrations ranging from 10^8^ CFU/mL to 10^3^ CFU/mL.

### 2.4 CO_2_ detection analysis

Carbon dioxide within the growth vials was measured before and after growth. Samples for gas analysis were arranged separately in 1 mL glass vials, drawn afterwards in heparinised syringes and analysed immediately using an ABL800 FLEX blood gas analyser (Radiometer, Denmark). Visible air bubbles were expelled immediately after collection via the syringes to avoid errors in pCO_2_, pO_2_ and saturated oxygen.

## 3 Results

Between 2016 and 2021, a total of 107 *A. schaalii* infections were isolated from 99 patients, 58 (59%) were men with a mean age of 59.5 (±6.1) years, and 41 (41%) were women with a mean age of 62 (±7.5) years.

Genitourinary samples were the most common, comprising 80.4% of all the isolates. The distribution of samples is described in Table 1.

**Table 1:** Samples where *A*. *schaalii* has been isolated.

Sample	N (%)
Urine	59 (55.1)
Seminal fluid	12 (11.2)
Surgically removed kidney stones	5 (4.7)
Female genital tract	5 (4.7)
Urethral exudate	4 (3.7)
Placenta (Chorioamnionitis)	1 (0.9)
Skin and soft tissue	14 (13.1)
Bone	5 (4.7)
Blood Culture	2 (1.9)

Half (7/14) of the skin and soft tissue infections were located in or near the inguinal region.

The five bone infections included 2 patients with chronic oteomyelitis, one in the tibia and the other in the fibula, 2 patients with a sacral infection, one with a sarcoma of the sacrum and the other with sarcoma of the femur.

Both bacteraemia were associated with UTI, one due to *A. schaalii* and the other of unknown origin.

### 3.1 UTI due to *A. schaalii*

UTI due to this microorganism accounted for 0.1% of all UTI in our hospital and 0.13% of UTI in patients over 60 years of age. In adults, 61.9% of the patients were male with a mean age of 74.4 (±15.2) while 38.1% were female with a mean age of 75.1(±18.2).

UTI due to *A. schaalii* was more frequent in men than in women: 0.18% vs 0.05% (χ^2^ = 24.4, df = 1, p < 0.0001).

Patients with UTI due to *A. schaalii* were older than those with a different pathogen isolated 66 (±6.7) vs. 58 (±0.2); p = 0.007.

*A. schaalii* was found as the only pathogen in 74.2% of UTI and 25.8% with other pathogens.

Isolation of *A. schaalii* was possible in 46 (78%) cases after growth in the Alfred 60/AST automated system and 91% had had bacterial counts > 10^5^ CFU/mL. The remaining 13 (22%) were cultured in a 5–10% CO_2_ atmosphere after being suspected following a Gram stain of the urine sample.

Patients with *A. schaalii* had a higher percentage of anatomo-functional alterations of the urogenital tract than those with UTI due to other microorganisms: 54.4% vs 37% (χ^2^ = 3.4, df = 1, p = 0.032).

For children studied, their mean age was 8.7(±5.3), with 5 of the 8 cases in the study having anatomo-functional alterations of the urogenital tract: two patients with bladder exstrophy and neurogenic bladder, one with neurogenic bladder, one with a kidney transplant and one with bilateral hydronephrosis and a kidney transplant.

### 3.2 Antimicrobial susceptibility

*A. schaalii* antimicrobial susceptibility is shown in Table 2. No isolate was susceptible to ciprofloxacin, whereas all were susceptible to penicillin, ampicillin, linezolid, vancomycin, teicoplanin, rifampicin and tetracycline.

**Table 2:** *A*. *schaalii* antimicrobial susceptibility.

Antimicrobial	Resistance n(%)	MIC criteria S	MIC criteria R
Penicillin	0(0)	≤0.125	>0.125
Ampicillin	0(0)	≤2	>8
Clindamycin	41(47.7)	≤0.5	>0.5
Erythromycin	36(46.8)	≤0.06	>0.06
Gentamicin	8(12.3)	≤0.5	>0.5
Tobramycin	9(15.3)	≤0.5	>0.5
Amikacin	5(8.3)	≤1	>1
Tetracycline	0(0)	≤2	>2
Vancomycin	0(0)	≤2	>2
Teicoplanin	0(0)	≤2	>2
Linezolid	0(0)	≤2	>2
Cotrimoxazole	28(36.4)	≤0.5	>0.5
Rifampicin	0(0)	≤0.06	>0.06
Ciprofloxacin	85(100)	≤0.25	>1

S, susceptible; R, resistant. Data in mg/L.

Fosfomycin CIM50 and CIM90 were 16 mg/L and 128 mg/L, respectively; while nitrofurantoin CIM50 and CIM90 were 32 mg/L and >512 mg/L, respectively.

No significant difference was found in antimicrobial susceptibility among isolates from ITU and from other specimens.

### 3.3 Colony Forming Units/mL detection assay

Alfred 60/AST yielded positive results for all *A. schaalii* with a concentration of 10^8^ CFU/mL, for 75% with a concentration of 10^7^ CFU/mL and for none of the rest.

### 3.4 CO_2_ detection analysis

The vials with enriched culture broth had a CO_2_ pressure of 12 mmHg (±1.9), with no significant difference being observed before and after growth.

## 4 Discussion

*A. schaalii* is a fastidious microorganism found in the microbiota of the genitourinary tract of healthy people. Data on infections due to this pathogen are scarce, with most reported cases being from UTI. Moreover, few reviews [20] concerning *A. schaalii* have been published, with most being a short series of cases, and ours being the most extensive to our knowledge, with 107 infectious episodes.

Our results showed that patients affected by *A. schaalii* were older, chiefly male and had underlying urinary tract conditions, especially in children; while patients with UTI were even older, both male and females. In addition, infections were produced chiefly in or near the genitourinary area, with more than half of the total being UTI as reported in other studies [8,9,20]. Nevertheless, the potential pathogenicity and virulence of this microorganism are demonstrated in our study by the 2 bacteraemia cases, the 5 bone and deep tissue infections and the chorioamnionitis case registered, which accounted for 7.5% of all the cases. Bone infections were related to open wound contamination or surgical procedures; indeed, three of them were associated to more pathogens. Deep tissue infections were likely linked to haematogenous seeding from a complicated UTI, as both bacteraemic episodes in our study were associated with UTI. In any case, virulence factors such as proteins required for pili assembly, the NanI gene encoding exo-alpha-sialidase and genes encoding heat shock proteins and type VII secretion system have been reported [21].

Remarkably, *A. schaalii* would not have been isolated in 77.6% of the UTI had we not used the Alfred 60/AST automated system. This is possible due to the fact that positive cultures are detected by laser nephelometry in subcultures grown in vials with a CO_2_-rich atmosphere. Positivity by this device allowed us to suspect UTI in cases that would have otherwise passed unnoticed. Indeed, UTI due to *A. schaalii* not processed by Alfred 60/AST was suspected only if a Gram stain was requested, which chiefly happens with urine samples from children or elderly people from the Emergency Room. Due to the large number of samples cultured in most microbiology laboratories, such a procedure in not feasible for all urine samples. Gram staining of these samples detected pyuria and Gram positive rods in high numbers, which led us to incubate subcultures in a CO_2_ atmosphere and easily isolate this pathogen. All this changed our workflow by adding a new sub-protocol aimed at detecting *A. schaalii*. Moreover, our experiments showed that Alfred 60/AST detects *A. schaalii* in concentrations over 10^6^ CFU/mL; thus excluding urinary tract colonisation cases. Our new work protocol observed bacterial growth after 24–48 h of incubation depending on the isolate; without this procedure, a work-up for "culture negative" UTI would have to be undertaken, wasting valuable time and effort and being detrimental for the patient.

Surprisingly, *A. schaalii* was found in five kidney stones. It is difficult to assess its role in the formation of the stone or how it was able to colonise it, but it is nonetheless an interesting field for more research.

Regarding antimicrobial susceptibility, our results showed the well-known resistance to ciprofloxacin, with also a high percentage of resistance to cotrimoxazole (36.4%). This should be further studied since natural resistance to trimethoprim in wild type *A. schaalii* has been described [18], but failure in the combination with sulfamethoxazole should imply the presence of acquired mechanisms. Other antimicrobials presented different levels of resistance; however, overall, β-lactams remain a good therapeutic option since no resistance to them was observed. Also, both fosfomycin and nitrofurantoin are good treatment alternatives, with a good MIC50, but should be further characterised. Moreover, antimicrobial resistance was similar regardless of the site of infection; perhaps supporting a common origin: i.e., the genitourinary tract, as supported by our results and other studies. However, one disadvantage was that we could not use susceptibility criteria designed specifically for *A. schaalii*; thus, more research is needed here.

All said, one of the strengths of our work is the large number of isolates included in the study, especially those from UTI. Nonetheless, mixed UTI including *A*. *schaalii* remain a challenge unless a Gram stain were performed to every positive urine sample. We hope that our work helps other laboratories to diagnose this pathogen.

In conclusion, we have presented a large series of *A. schaalii* infections. Although it is not a mainstream pathogen, -it mainly affects elderly patients or those with underlying urinary tract conditions -it is hard to isolate in routine urine sample cultures and is resistant to commonly used empirical antimicrobials. These properties make *A. schaalii* infections difficult to treat and prone to persistence and recurrence. Therefore, awareness about this microorganism should be taught among physicians and microbiologist alike.

## Declarations

This study is part of a project reviewed and approved by the IIS La Fe Ethics Committee.

(Ref. 2015/0296, June 2018).

## Data availability statement

Data associated with this study have not been deposited in a publicly available repository, but are freely available upon request from the authors and approval by the IIS La Fe Ethics Committee.

## CRediT authorship contribution statement

**J.M. Sahuquillo-Arce:** Writing – original draft, Supervision, Methodology, Investigation, Formal analysis, Data curation, Conceptualization. **P. Suárez-Urquiza:** Writing – original draft, Methodology, Formal analysis, Data curation. **A. Hernández-Cabeza:** Writing – review & editing, Investigation, Conceptualization. **L. Toffan:** Writing – review & editing, Investigation. **R. Chouman-Arcas:** Writing – review & editing, Investigation. **M. García-Hita:** Writing – review & editing, Investigation. **O. Sabalza-Baztan:** Writing – review & editing, Investigation. **A. Sellés-Sánchez:** Writing – review & editing, Methodology, Investigation. **N. Lozano-Rodríguez:** Writing – review & editing, Investigation. **J. Martí-Cuñat:** Investigation. **J.L. López-Hontangas:** Writing – review & editing, Resources, Project administration, Funding acquisition.

## Declaration of competing interest

The authors declare that they have no known competing financial interests or personal relationships that could have appeared to influence the work reported in this paper.
